# Organization of St. Louis Veterinarians

**Published:** 1897-06

**Authors:** 


### ORGANIZATION OF ST. LOUIS VETERINARIANS.

THE St. Louis veterinarians held a meeting and organized a city association in the Board of Education building in that city. Among those present were Drs. Hammerstein, Piatt, Smedberg, Sheridan, Dickey, Kelly, Menisterno, Ellis, Kammerer, Hemplemann, Crowley, James, Cass, and Day. A Constitution, By-Laws, and Code of Ethics were adopted. The following officers were elected: President, Charles V. Ellis; Vice-President, A. J. Hammerstein; Secretary, R. A. Kammerer; Treasurer, H. B. Piatt.

